# Protein Kinases: Potential Drug Targets Against *Schistosoma japonicum*

**DOI:** 10.3389/fcimb.2021.691757

**Published:** 2021-07-01

**Authors:** Kaijuan Wu, Xingyu Zhai, Shuaiqin Huang, Liping Jiang, Zheng Yu, Jing Huang

**Affiliations:** ^1^ Department of Parasitology, School of Basic Medical Science, Central South University, Changsha, China; ^2^ China-Africa Research Center of Infectious Diseases, Central South University, Changsha, China; ^3^ Department of Microbiology, School of Basic Medical Science, Central South University, Changsha, China

**Keywords:** schistosomiasis, *Schistosoma japonicum*, protein kinases, drug targets, kinase inhibitors

## Abstract

*Schistosoma japonicum* (*S. japonicum*) infection can induce serious organ damage and cause schistosomiasis japonica which is mainly prevalent in Asia and currently one of the most seriously neglected tropical diseases. Treatment of schistosomiasis largely depends on the drug praziquantel (PZQ). However, PZQ exhibits low killing efficacy on juvenile worms and the potential emergence of its drug resistance is a continual concern. Protein kinases (PKs) are enzymes that catalyze the phosphorylation of proteins and can participate in many signaling pathways *in vivo*. Recent studies confirmed the essential roles of PKs in the growth and development of *S. japonicum*, as well as in schistosome-host interactions, and researches have screened drug targets about PKs from *S. japonicum* (SjPKs), which provide new opportunities of developing new treatments on schistosomiasis. The aim of this review is to present the current progress on SjPKs from classification, different functions and their potential to become drug targets compared with other schistosomes. The efficiency of related protein kinase inhibitors on schistosomes is highlighted. Finally, the current challenges and problems in the study of SjPKs are proposed, which can provide future guidance for developing anti-schistosomiasis drugs and vaccines.

## Introduction

Schistosomiasis is a serious neglected tropical disease that causes a series of pathological damages to humans. In 2019, the World Health Organization (WHO) showed that preventive chemotherapy (PC) for schistosomiasis was required in 51 countries with a total of 236.6 million people (WHO, 2020). Schistosomiasis is caused by mainly three species including *Schistosoma japonicum*, *Schistosoma mansoni* and *Schistosoma hematobium*. As the causative agent of hepato-intestinal schistosomiasis, *S. japonicum* is mainly prevalent in southern China, to a lesser extent Indonesia and the Philippines. This parasite has a complex life cycle, which involves two hosts including molluscan and mammalian hosts, and six developmental stages including adult worm, ovum (egg), miracidium, sporocyst (mother sporocyst and daughter sporocyst), cercaria, and schistosomula. Cercariae escape from *Oncomelania hupensis*, encounter humans or other mammals in the infested water, penetrate their skins to form juvenile worms, and migrate through the lungs to the mesenteric blood vessels, where they mature into the adult worms and lay eggs. Most eggs flow into the liver, while others are excreted with feces, hatch in water, and then burrow into snails to become cercariae (Zhang et al., 2020). Praziquantel (PZQ) is the most efficient drug used for the treatment of schistosomiasis so far, whose treatment cost is commonly less than US$1 per patient in developing countries (Lo et al., 2016). However, PZQ still has some problems such as low solubility, side effects, low efficacy against juvenile worms of the parasite and its unknown biological target. More importantly, previous studies confirmed that the PZQ resistance existed in *S. japonicum* under continuous drug pressure (Li et al., 2011; Liang et al., 2011). Therefore, it is essential to develop a second generation of anti-schistosomal drugs.

Protein kinases (PKs) play important roles in the regulation of various cell functions such as cell proliferation, differentiation, and apoptosis. These processes are caused by catalyzing protein phosphorylation which transmits the main intracellular and extracellular signals to the specific cell even its nucleus and transfers the phosphate groups from ATP to the protein substrate (Manning et al., 2002; Cheetham, 2004). It has been reported that PKs, the important drug targets of many carcinomas, are considered targets against parasitic diseases (Boyle and Koleske, 2007; Beckmann et al., 2012). Since the importance of PKs in the growth and development of *S. mansoni*, the major helminth species, was confirmed, a large number of studies on PKs of *S. mansoni* have been carried out. Many researches have shown that PKs were associated with oxidative stress, tegument, development, maturation, and survival of *S. mansoni* (Avelar et al., 2019; Gava et al., 2019). A recent study uncovered two new protein kinases (TAO and STK25) which collaborate to maintain muscle-specific messenger RNA transcription in *S. mansoni*, and were expected to be reliable targets (Wang et al., 2020). Although the phosphorylation analysis shows that PKs are important for the growth and development of *S. japonicum* (Luo et al., 2012), the research on SjPKs is not as comprehensive as that on *S. mansoni*. Recently, with the application of a new computational pipeline, several new targets and drugs have been identified as starting points in the development of new schistosomicidal agents, many of which are kinases and kinase inhibitors (Giuliani et al., 2018). These discoveries indicate that PKs have the potential to be the drug targets against schistosomiasis. In this review, we will present an overview of SjPKs from many aspects including classification, different functions and related kinase inhibitors, compared with other kinds of schistosomes, which aim to elucidate the essentiality of PKs in physiological function of *S. japonicum*. We also analyze the limitations and problems of current research on SjPKs. The ultimate goal is to encourage more relevant researches and provide more references for the invention of anti-schistosomiasis drugs and vaccines in the future studies.

## Phosphoproteomics Reveals the Important Role of PKs in *S. japonicum*

Through the technology of microvolume immobilized metal-ion affinity chromatography (IMAC) pipette tips coupled to nanoLC–ESI-MS/MS, *in vivo* proteins phosphorylation sites have been performed in different life stages of *S. japonicum* and subsequently 127 phosphorylation sites in 92 proteins have been characterized in *S. japonicum* (Luo et al., 2012). The distribution of phosphorylations among serine, threonine, and tyrosine is 91%:7%:2%, which is similar to the distributions observed in higher eukaryotes (Molina et al., 2007). Some important PKs are discovered in this experiment, such as thymidylate kinase, cAMP-dependent protein kinase type II regulatory subunit, and cell division protein kinase 10, revealing that these kinases might be involved in important signaling pathways in *S. japonicum* (Luo et al., 2012). However, the further confirmation experiments of the phosphorylation sites and PKs were hindered owing to the lack of specific antibodies. Subsequently, researchers, made an attempt to apply petitanium dioxide (TiO2) based phosphoproteomic method to characterize phosphoproteins in schistosomula, adult females, and adult males of *S. japonicum* (Cheng et al., 2013). The results showed that the distribution of phosphorylations among serine, threonine, and tyrosine was 90.1%:9.4%:0.5% in *S. japonicum*. Many pivotal SjPKs were also observed in this method. Heat shock protein 90 (Hsp90) might regulate PKs including glycogen synthase kinase 3 (GSK3) and cell division cycle 37 homologue (Cdc37), which is essential in regulation of development in *S. japonicum* (Cheng et al., 2013). Although there are still technical limitations, these studies indirectly demonstrate that it may be feasible to find measures to interfere with schistosomiasis japonica through PKs.

The importance of PKs has also been noted in other species of schistosomes, one example being *Schistosoma mekongi* (*S. mekongi*). A study explored the effect of PZQ on *S. mekongi* proteome and phosphoproteome, in which it was found that proto-oncogene tyrosine-protein kinase Src was mainly associated with the alteration of phosphorylation for PZQ response by kinase–substrate predictions (Chienwichai et al., 2020). Therefore, applying Src kinase inhibitors could be a novel approach to treat Mekong schistosomiasis. In another study on *S. mansoni*, the phosphoproteome of *S. mansoni* was first comprehensively analyzed based on a novel peptide kinomic array, which has characterized 12936 phosphorylation sites in 3176 proteins (Hirst et al., 2020). The phosphorylation sites identified from *S. mansoni* cover a wider area than these identified from *S. japonicum*. It was also shown that many PKs such as CaMKII, PKA and CK1/2 are of great importance to schistosome function (Hirst et al., 2020). Interestingly, the distribution of phosphorylations among serine, threonine, and tyrosine is 68%:20%:12% in *S. mansoni*, and a greater proportion (3 to 4-fold) of phosphotyrosine is determined compared to other organisms (Rigbolt et al., 2011; Lasonder et al., 2012). These findings suggest that protein tyrosine kinases may play a more significant regulatory role in *S. mansoni* than in many other eukaryotes. Therefore, a more comprehensive phosphorylation analysis of *S. japonicum* needs to be performed to explain this discrepancy. In short, PKs play central regulatory roles in *S. japonicum*, but the number and specific function are unknown.

## The Classification of PKs in Schistosomes

According to the structure of PKs, they can be classified into eukaryotic protein kinases (ePKs) and atypical protein kinases (aPKs). Most of them belong to ePKs family. aPKs lack conserved eukaryotic kinase motifs compared with ePKs. Based on solely catalytic domain sequences, ePKs can be classified into nine large groups, including AGC (cAMP-dependent PKs/protein kinase G/protein kinase C), CAMK (Calcium/calmodulin-regulated kinases), CMGC (Cyclin-dependent kinases and relatives), TK (Conventional Protein-Tyrosine Kinase Group), CK1 (Cell kinase I), STE (MAP kinases), RGC (Receptor guanylate cyclases), TKL (Tyrosine kinase-like), and Other (Roskoski, 2015). There are 252 ePKs in the kinome of *S*. *mansoni* including nine eukaryotic groups (AGC, CK1, STE, CAMK, CMGC, RGC, TK, TKL, and Other), which account for ~2% of the entire encoded proteins in the parasite genome. Among them, approximately 15% of the functional mechanisms have been elucidated (Andrade et al., 2011; Grevelding et al., 2018). A total of 261 ePKs are identified in *S. haematobium* and can be classified into several groups (CAMK, AGC, TK, STE, TKL, CK1, RGCs, CMGC, and Other). Compared to the kinome of *S*. *mansoni*, they have a highly overall sequence identity (82–92%), similarity (87–94%) and a relatively conserved length (0–7% difference) between pairs of kinases (Stroehlein et al., 2015). In *S. japonicum*, the latest research has shown that 222 PKs in *S. japonicum* can also classified into nine major ePKs groups (AGC, CK1, STE, CAMK, CMGC, RGC, TK, TKL, and Other) (Giuliani et al., 2018).

**Figure 1:**
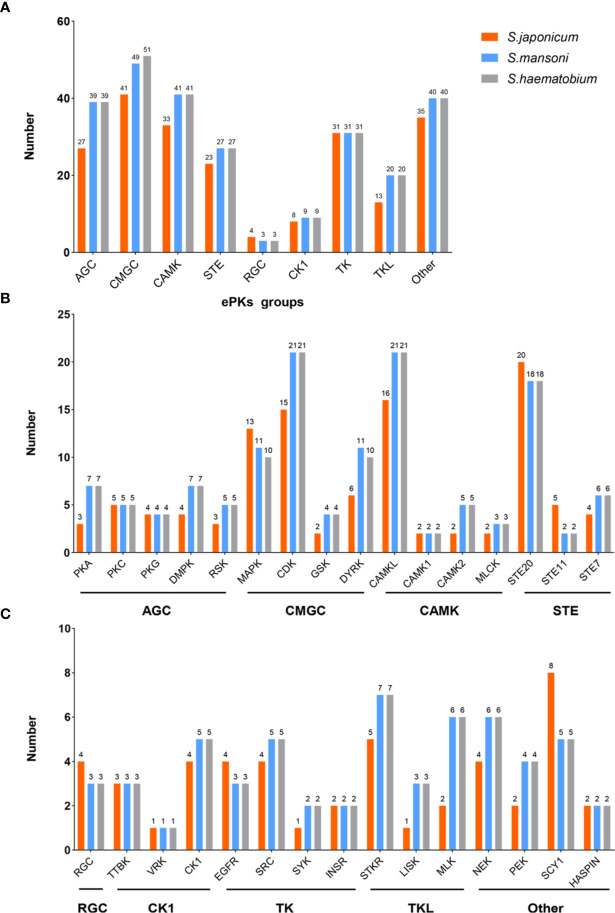
The comparison of ePKs groups and main families of three species of schistosomes. **(A)** The classifications of ePKs groups among three schistosomes including AGC, CMGC, CAMK, STE, RGC, CK1, TK, TKL, Other. **(B)** The classifications of ePKs main families in different groups (AGC, CMGC, CAMK, STE). **(C)** The classifications of ePKs main families in different groups (RGC, CK1, TK, TKL, Other).

Only from the comparison of numbers (**Figure 1**), the ePKs groups of *S. mansoni* have high similarity with *S. haematobium*. However, *S. japonicum* has less ePKs than them in most groups and families. At present, there is no research focusing on analyzing the homology and similarity between the protein kinome of *S. japonicum* and other species. Therefore, it is difficult to directly get accurate *S. japonicum* kinome information according to other reported species of schistosome kinomes, although these data can be referenced. This also enlightens us to obtain comprehensive information about the protein kinome of *S. japonicum* as soon as possible.

## The Functions of Different PKs in *S. japonicum*

Since the middle of the last century, the function of schistosome kinases has attracted much attention. PKs-mediated signaling pathways regulate the physiological and morphological changes of various organisms. Their evolutionary conservation has laid a good foundation for functional research between different organisms. Recently, with the rise of research on PKs of *S. japonicum*, more and more studies have shown that protein kinase plays a key role in the biological activities of *S. japonicum*. In **Table 1**, we summarized the SjPKs and their functions based on previous studies.

**Table 1:** The characteristics of protein kinases in *Schistosoma japonicum*.

Protein Kinase	Classification	The Amino acid sequence similarity with human	Location	Expression	Function	Reference
Epidermal growth factor receptor (EGFR)	TK	30%	Tegument and intestines	All developmental stages	Advance worms growth	(Xiang et al., 2020)
Insulin receptor 1 (IR1)	TK	40.8%(TK domain)	Tegument, muscles and the intestinal epithelium	Schistosomulum, males and females	Advance worms growth and development	(You et al., 2010)
Insulin receptor 2 (IR2)	TK	44.6%(TK domain)	Vitelline and parenchyma	Males and females	Participate in worms sexual maturation	(You et al., 2010)
Tyrosine kinase 4 (TK4)	TK	21.3%	Spermatocytes and oocytes	All developmental stages, males > females	Advance worms growth and development	(Ding et al., 2017)
Tyrosine kinase 3 (TK3)	TK	unknown	Unknown	Mainly eggs, females > males	Advance worms growth and eggs production	(Wu, 2018)
Glycogen synthase kinase 3β (GSK3β)	CMGC	unknown	Unknown	Mainly eggs, males > females	Maintain worms survival	(Liu et al., 2020)
Ca^2+^/calmodulin-dependent protein kinase II (CAMKII)	CAMK	81%	Unknown	Unknown	Enhance L-type Ca^2+^ channels	(You et al., 2013)
Extracellular signal-regulated kinases (ERKs)	CMGC	unknown	Unknown	Mainly Cercariae, females > males	Advance worms growth and development	(Wang et al., 2006)
c-Jun amino-terminal kinases (JNKs)	CMGC	unknown	Unknown	Mainly females	Participate in female sexual maturation	(Wang et al., 2006)
Right open reading frame protein kinase 2 (Riok-2)	aPKs	52%	Vitellarium and ovary	Mainly females and eggs	Participate in female sexual maturation	(Zhao et al., 2017)

## TK Family May Play an Important Role in the Survival of *S. japonicum*

There are two main subdivisions in the ePKs superfamily: the protein-serine/threonine kinases and the protein-tyrosine kinases (Hanks and Hunter, 1995). It has been reported that the activity of TK family is central to many cellular processes such as cell adhesion, migration, proliferation, differentiation as well as the initiation and maintenance of inflammation (Hubbard and Till, 2000; Ishizawar and Parsons, 2004; Page et al., 2009). TK family consists of receptor tyrosine kinases (RTKs) and non-receptor or cytosolic tyrosine kinases (CTKs). RTKs, as one of the transmembrane receptors, are characterized by the inherent tyrosine kinase activity. They are typically composed of an extracellular domain that can bind to specific ligands, which enable change of the protein structure and allow the intracellular kinase domain to catalyze the phosphate group into the tyrosine residues in itself. RTKs are expressed in tissues over the body during intrauterine development and in adulthood, which regulates cell differentiation, proliferation, survival, and metabolism (Moshirfar et al., 2020; Wintheiser and Silberstein, 2020). CTKs can relay the transduction of signals from extracellular receptors including cytokine receptors, immunoglobulin receptors, and other signaling pathways, which form signal transduction complex *via* tyrosine phosphorylation, activating the downstream signal transduction (Gocek et al., 2014).

**Figure 2:**
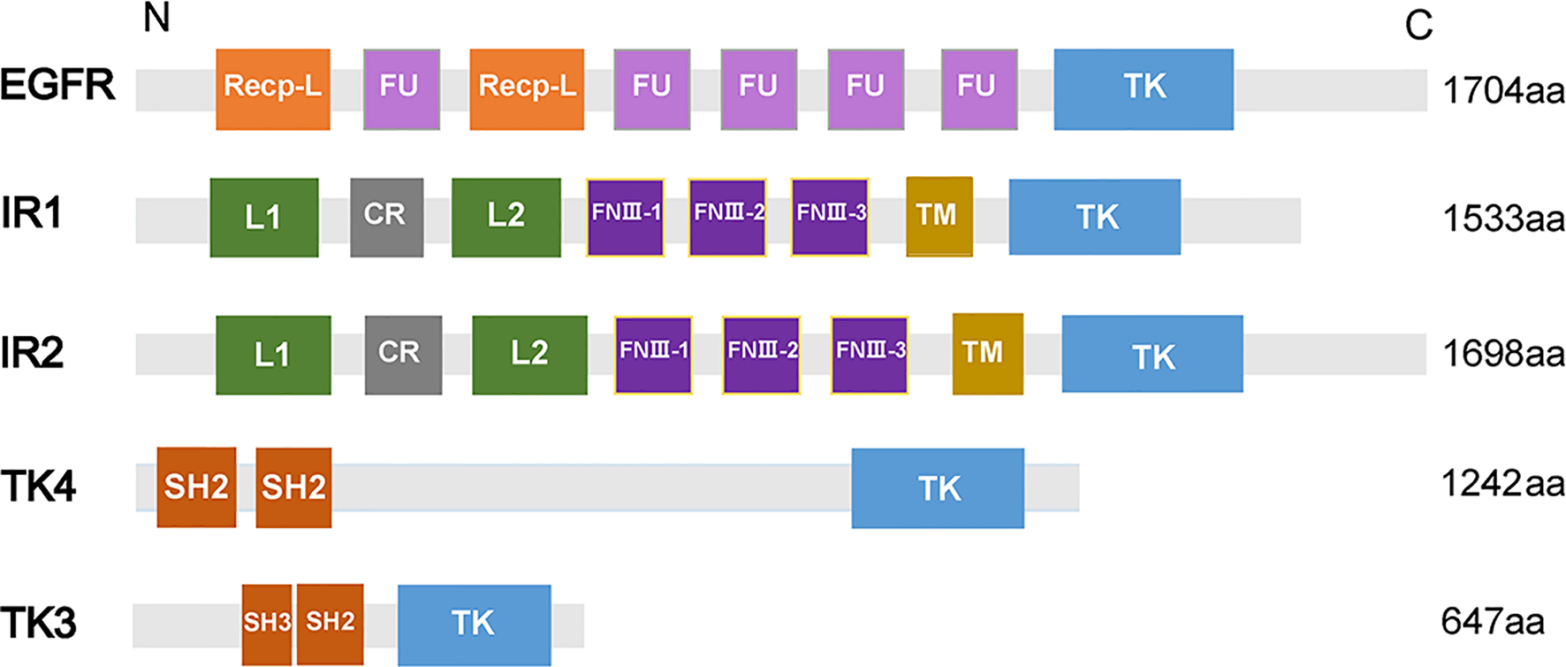
Domain structures of PTKs family. The domain structures including Receptor L domain (Recep-L), Furin-like repeats (FU), ligand binding loop 1 (L1), ligand binding loop 2 (L2), Cysteine-rich region (CR), Fibronectin type III domain (FnIII-1, FnIII-2, FnIII-3), Transmembrane domain (TM), Src homology domain (SH2, SH3), Tyrosine kinase domain (TK). The lengths of amino acid sequences and domain structures of different PTKs are different.

Epidermal growth factor receptor (EGFR), a transmembrane glycoprotein, is a member of RTKs (Grapa et al., 2019). EGFR combines with epidermal growth factor (EGF) and then is activated to regulate cell growth and differentiation (Gamou and Shimizu, 1992). In humans, EGFR is correlated with the pathogenesis and progression of different carcinoma types (Normanno et al., 2006). In *S. mansoni*, an EGFR homolog called SER is expressed predominantly in the muscle of adult male and female worms, elucidating that SER may participate in muscle development (Ramachandran et al., 1996). In addition, SER is able to bind to human EGF to trigger Ras/MAPK pathways in the cell (Vicogne et al., 2004). A homolog of EGFR was identified from *S*. *japonicum* (SjEGFR) in the latest research, and the transcript of the *SjEGFR* was expressed in the tegument and in the intestines of *S. japonicum*. SjEGFR can be detected in different growth stages of schistosomes. The amino acid sequence of SjEGFR is 85% homologous with SmEGFR, which contains a tyrosine kinase, catalytic domain, receptor L domain, and some Furin-like repeats (**Figure 2**) (Xiang et al., 2020), indicating that SjEGFR may have similar functions with SmEGFR.

The research also reported that knockdown of *SjEGFR* can suppress the growth of worms, showing the decreasing number of spermatogonia and spermatocytes in the smaller testis of male worms and immature oocytes in the narrow-shaped ovary of female worms, which was consistent with the results of *SmEGFR* (Xiang et al., 2020). However, no significant effects on female *S. japonicum* have been detected after knockdown of *SjEGFR* by RNAi technology. The abnormal appearance of female gonad may result from the inhibition of male growth interfering with the development of female growth. In brief, SjEGFR possibly regulates the survival and development of *S. japonicum*, which relies on promoting male maturity. The specific mechanism requires further study on its downstream pathways.

**Figure 3:**
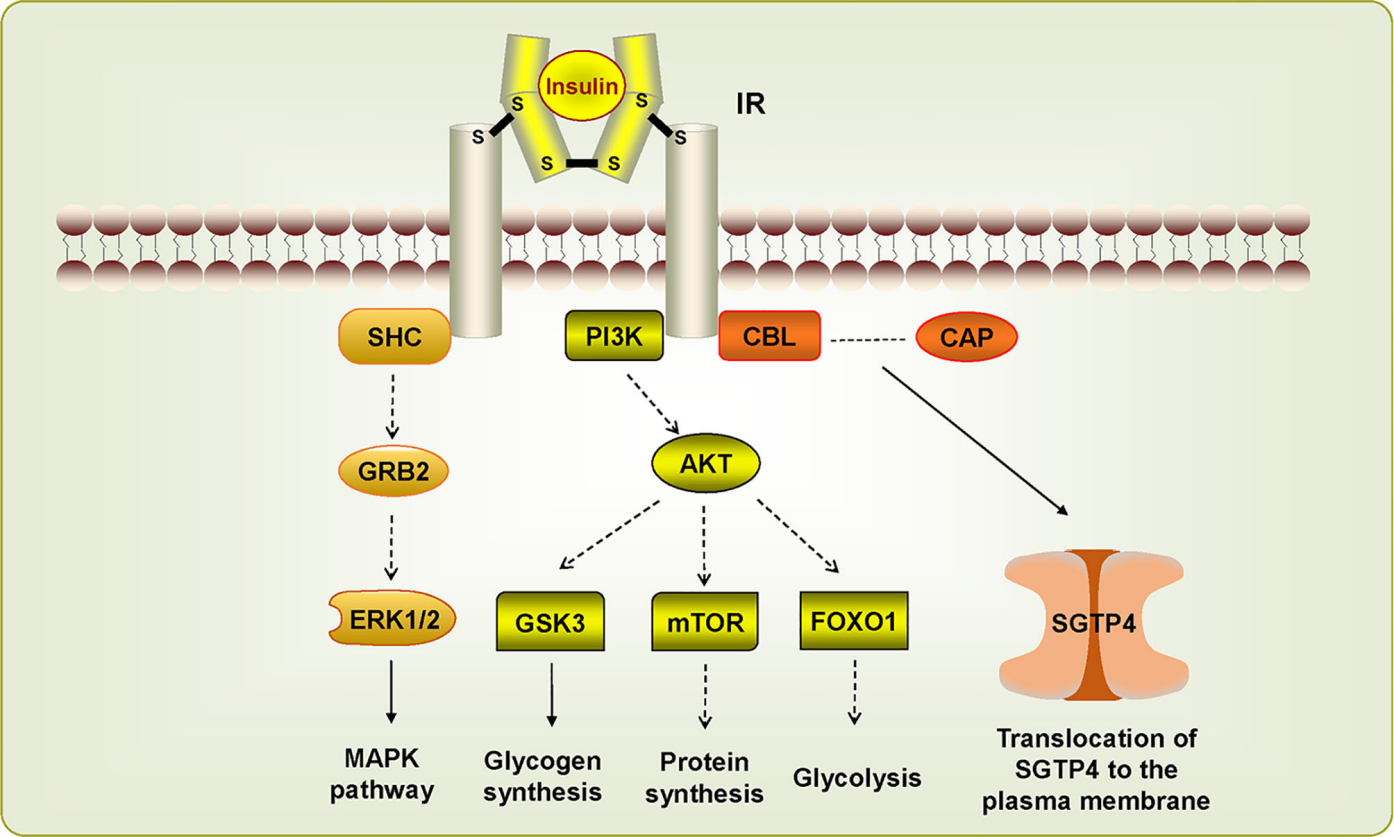
Insulin receptor signaling pathways in *Schistosoma japonicum*. The dotted line suggests this pathway has not (yet) been demonstrated in *S. japonicum*. The IR signaling pathways are activated by insulin, which stimulate downstream pathways. SHC can bind IR to induce MAPK pathway. PIK3/AKT pathway is associated with glycogen synthesis, protein synthesis, and glycolysis. CBL/CAP pathway is involved in the translocation of SGTP4 to the plasma membrane.

Insulin receptor (IR), another member of RTKs, can be activated by insulin, which regulates glucose transport, lipid synthesis, storage, and mobilization in humans (Ward and Lawrence, 2009; Haeusler et al., 2018). A homolog of IR was first discovered in *S. japonicum* by genome analysis, whereas no insulin growth factor (IGF) or insulin molecules were found (Hu et al., 2003). It has been confirmed that host insulin can be utilized by adult *S. japonicum* and it is involved in important metabolic pathways including glucose metabolism, phosphoinositide-3-kinase (PI3K) pathway, and MAPK pathway (You et al., 2009). The results of the yeast two-hybrid experiment suggests that two types of IRs isolated from *S. japonicum* (SjIR-1 and SjIR-2) can specifically bind to human insulin. They show 70% and 74% similarity to SmIR-1 and SmIR-2, respectively. Two conserved domains (TK and LD domain) and insulin binding site are located in L1 sub-domain (**Figure 2**) (You et al., 2010). SjIR-1 is predominantly expressed in the tegument basal membrane, muscles and the intestinal epithelium of worms, and SjIR-2 is expressed in the vitelline tissue of female worms and the parenchyma of males, indicating their possible functions in the regulation of growth and development (You et al., 2010). Knockdown of *SjIR1* or *SjIR2* can downregulate the main downstream genes in the insulin signaling pathway such as *PI3K*, glycogen synthase (*GYS*), and so on (You et al., 2015). Meanwhile, double knockdown of both SjIR1 and SjIR2 is more effective. SHC transforming protein 3 (*SHC*) and CBL E3 ubiquitin protein ligase (*CBL*) are also found to be downregulated after the knockdown of SjIR1 or SjIR2. In humans, SHC can be activated by the combination of IRs and insulin, and *CBL* is phosphorylated by IRs to participate in the insulin signaling pathway. The appearance of *SHC* and *CBL* by knockdown of *SjIR1* or *SjIR2* illustrates a similar insulin signaling pathway in *S. japonicum* and humans. In addition, the expression of SGTP4 and SGTP1 are significantly upregulated when either *SjIR1* or *SjIR2* was knocked down, which is consistent with the previous study (You et al., 2010; You et al., 2015). The predicted insulin receptor signaling pathways based on insulin KEGG pathway (www.chgc.sh.cn/japonicum/Sjpathway) and current studies are shown in **Figure 3**. Recently, insulin-like peptides (ILPs) were found in schistosomes by genome-wide searches (Wang et al., 2014). ILPs also have a basic insulin structure composed of an A peptide and a B peptide linked by disulfide bridges and share a similar structure with SmILPs. In addition, the combination of SjILPs and SjIRs can activate the Erk/MAPK sub-pathway in schistosomes, which is better than the combination of insulin and SjIRs. Coincidentally, SjILPs are also located in the tegument and muscles of worms, vitelline tissue of female worms and the parenchyma of males worms, which are similar to the location of SjIRs, indicating that SjILPs may interplay with SjIRs to regulate insulin signaling (You et al., 2010; Du et al., 2017). The expression of SjILPs in eggs, miracidia, and female worms is comparatively higher than in schistosomula, cercariae, and male worms in the latest study, which demonstrates that SjILPs not only can activate the parasite insulin pathway, but also can be involved in other signaling pathways in this parasite such as eggs hatching and movement of miracidia (Du et al., 2019). Knockdown of *SjILPs* causes declination in the level of glucose consumption of worms, suggesting the binding of SjILPs and SjIRs also participates in glucose uptake of *S. japonicum* (Du et al., 2019).

SjLD2 encoding the L1, CR, and L2 sub-domains (R37-C115) of SjIR2 has been designed as a vaccine candidate (rSjLD2) against *S. japonicum*. rSjLD2 can significantly reduce the fecal eggs and stunt the worms by inhibiting the binding of human insulin to SjIR-2 (You et al., 2010; You et al., 2012). Another study also shows that rSjLD1 and rSjLD2 are able to induce a significant growth defect of worms and contribute to decreasing intestinal granuloma density (You et al., 2015). In order to improve the vaccine efficacy of SjIR, a study sought to combine rSjIR with *S. japonicum* triose-phosphate isomerase (SjTPI), conjugated with two different adjuvants (QuilA and montanide ISA 720VG) (You et al., 2018). Nevertheless, the rSjTPI-rSjLD1 combination did not increase the worm reduction rate and led to a lower level of protection. rSjLD1 formulated with either of two adjuvants can enhance the level of protection and enable the immune system to elicit mainly Th1 response against schistosome infection (You et al., 2018). These studies suggest that, to develop safer and more effective drugs or vaccines, it is vital to further explore the functions of these proteins and the relationship between the host and the parasite.

TK4, which belongs to the spleen tyrosine kinase (Syk) family of CTK kinases, was found in *S. mansoni* and it may play an important role in the development of gonad because main signals could be detected in the oocytes and the spermatocytes, and thus TK4 could become a new target for interfering transmission and disease expansion (Knobloch et al., 2002; Beckmann et al., 2010). It has been reported that SmTK4 can regulate the proliferation and/or differentiation of germinal cells by activation of a MAPK pathway (Beckmann et al., 2010). A study shows that the conserved gene sequence of *S. japonicum* TK4 (SjTK4) is 98% identical to the sequence of SmTK4, and there are two SH2 domains and one TK domain in SjTK4 (**Figure 2**). The level of mRNA in male schistosome is significantly higher than that in female and significant signals presented in spermatocytes and oocytes, elucidating that the expression of TK4 is related to the gender and germ cell development of *S. japonicum* (Zang et al., 2014). Later, it was discovered that the SjTK4 amino acid sequence shows 76.9% identity and 82.7% similarity with SmTK4 sequence, the gene is differentially expressed in all developmental stages of schistosome both in females and males. With the therapy of piceatannol (a Syk kinase-specific inhibitor), many morphological changes were observed in the testes and ovaries of *S. japonicum*, and the size and number of whole germ cells in both the testis and ovary are smaller and fewer than the control group, suggesting that TK4 may have a significant effect on the production and development of eggs *in vitro* (Ding et al., 2017). In addition, the application of piceatannol significantly upregulates the expression of genes related to egg-shell production including P14, P48, egg-shell precursor, and FS800, which in agreement with the results in imatinib-treated *S. mansoni*, further indicating the functional similarity between SjTK4 and SmTK4 (Buro et al., 2014).

TK3 is a member of the Src family, which has an essential function in signaling transduction pathways constituting the cytoskeleton in the gonads of *S. mansoni* (Kapp et al., 2004). The results of the yeast two-hybrid experiment shows that the upstream interacting molecules with SmTK4 are SmTK3 and SmTK6, and the tandem SH2-domain of SmTK4 can interact with SmTK3 (Beckmann et al., 2010). In *S. japonicum*, the latest study reveals that the SjTK3 amino acid sequence is 91.6% homologous with that of SmTK3, which contains one SH3 domain, one SH2 domain, and one TK domain, the high sequence similarity indicated that SjTK3 may have a similar functional mechanism to SmTK3. TK3 is differentially expressed in different growth stages of schistosomes, with the highest expression level found in egg. The expression level gradually decreases from cercariae to juvenile worms, and increases in the adult stage, and its expression in females is significantly higher than that in males. The numbers of eggs, spermatogonia and mature oocytes are reduced after treatment by a Syk kinase-specific inhibitor, indicating that TK3 may play a role in maintaining schistosome reproductive development and laying eggs *in vitro* (Wu, 2018). In addition, the upregulation of P14, P48, and FS800 also suggests that SjTK3 may activate SjTK4 to regulate the development of eggs.

Therefore, four members of TK family are found to be involved in various physical processes of *S. japonicum*. Amino acid sequences of SjEGFR, SjTK4 and SjTK3 are highly homologous with the sequence of SmEGFR, SmTK4 and SmTK3, suggesting that the functions of the members of TK group in *S. japonicum* and *S. mansoni* PKs may be similar. It may be a possible way to explore unknown TK group of PKs in *S. japonicum* based on the identified PKs in *S. mansoni*. After all, SmPKs are currently the most thoroughly studied PKs.

## Glycogen Synthase Kinase 3 May Be Involved in The Main Signaling Pathway in *S. japonicum*

Glycogen synthase kinase 3 (GSK3), a member of the CMGC group, participates in a host of signaling pathways. The activity of GSK3 is primarily involved in Wnt–β-catenin signaling pathway, which plays an important role in inhibiting β-catenin nuclear translocation by the activity of a destruction complex including the tumor suppressors AXIN1, adenomatosis polyposiscoli (APC), the kinases casein kinase 1 (CK1) and GSK3β (Bugter et al., 2021). β-catenin nuclear translocation activates T-cell factor/lymphoid enhancer factor (TCF/LEF) to control the expression of target genes, and thus, GSK3 can act as a tumor suppressor by restricting canonical Wnt–β-catenin signaling (Clevers and Nusse, 2012; Benoit et al., 2014). Meanwhile, Wnt–β-catenin signaling pathway regulates complex normal cellular processes such as cell differentiation and development (Sharma and Pruitt, 2020). GSK3 has been shown to influence related biological functions in many organisms, for example, the knockdown of *Haemaphysalis longicornis GSK3β* can cause a significant decrease in reproduction and abnormalities in eggs and hatching (Rahman and You, 2019). Other studies also have shown that it can be also associated with parasitic diseases such as *Toxoplasma gondii*, *Plasmodium falciparum*, *Leishmania donovani* (Qin et al., 1998; Xingi et al., 2009; Masch and Kunick, 2015). In addition, a previous study suggests that there exists a Wnt signaling pathway in the *S. japonicum* genome (Berriman et al., 2009).

In schistosome, GSK3 was identified to exist in the schistosome kinase genome. Surprisingly, GSK (Sjp_0026020) is regarded as a candidate target for anti-schistosomiasis (Luo et al., 2012; Giuliani et al., 2018). *S. japonicum* GSK3β (SjGSK3β) is an isoform of GSK3 (Rayasam et al., 2009), its amino acid sequence shows 39.61% homology with human. Recent research shows that SjGSK3β can be expressed in different life cycle stages, particularly in the egg stage and higher in male worms than that in female worms (Liu et al., 2020). Suppression of SjGSK3β activity can affect the cell activity and survival of schistosomes after applying GSK inhibitor (CHIR-99021) or siRNA-88. The results of the yeast two-hybrid library confirm the interacting partners of SjGSK3β such as Cdc37, 14-3-3 protein, tegument antigen (I(H)A), and V-ATPase proteolipid subunit (Liu et al., 2020). In *S.japonicum*, how SjGSK3β participates in the Wnt–β-catenin pathway and how SjGSK3β, together with its interacting partners, co-regulate other signaling pathways are unknown and need to be further explored.

## CAMKII Is Related to Calcium Homeostasis in *S. japonicum*

Currently, PZQ is the only effective drug against three major schistosomes, the mechanism of action is not yet clear, but many studies have shown that it is closely associated with calcium homeostasis (Doenhoff et al., 2008). It has been illustrated that Ca^2+^/calmodulin-dependent protein kinase II (CAMKII) can enhance L-type Ca^2+^ channels by phosphorylating both α and β subunits of voltage-gated Ca^2+^ channels (Grueter et al., 2008). An article on the mechanism of PZQ found that CAMKII was significantly up-regulated in mice infected with *S. japonicum* that were given a certain dose of PZQ, and then by knocking out CAMKII genes, it was observed that the activity of adult worms declined. Instillation of IC50 (IC50 = 0.83 nM) PZQ into CAMKII knockout mice shows that the activity of adults is lower from 47-61% to 23-27% than that of wild-type (WT) mice, indicating that inhibition of CAMKII can enhance the efficacy of PZQ. The increase in transcription of CAMKII in *S. japonicum* suggests that it can act as a response molecular to up-regulate Ca^2+^ levels, a known event in PZQ action (You et al., 2013). Moreover, the level of calmodulin bound and activated by released Ca^2+^ to involve in calcium pathway is strongly increased in both female worms and male worms, which suggests it is an important component of calcium signaling in response to PZQ treatment (You et al., 2013). In mammals, calmodulin has been demonstrated to activate CAMKII by binding two Ca^2+^ ions (Shifman et al., 2006). In *S. japonicum*, a similar calcium pathway may also exist, underlaying future research about the interaction between CAMKII and schistosomes. Another study applied selective and nonselective CAMK/Kinase inhibitors including 1Naphthyl PP1 (1NAPP1) and Staurosporine (STSP) in schistosomes with or without the presence of PZQ *in vivo* and *in vitro* (Nawaratna et al., 2020). The results showing that the combination of PZQ and 1NAPP1 treatment is more effective to kill 7-day old schistosomula and weaken the motility of adult worms (Nawaratna et al., 2020). However, only the group treated with the highest dose of STSP conjunction with PZQ exhibits the highest decrease in worms and eggs compared with the control group and thus significantly lower (58%) liver egg counts compared with the PZQ group *in vivo* (Nawaratna et al., 2020).

In summary, although there is no specific elucidation of the role of CAMKII in the physiological process of schistosomes, it is necessary to confirm the functional effects of CAMKII inhibitors on calcium homeostasis in schistosomes because they have the potential to become adjuvant drugs for PZQ in the treatment of patients infected with schistosomiasis. However, CaMKII in schistosome has 81% homology with human CaMKII, so it is necessary to design CaMKII-related drugs more specifically (You et al., 2013).

## MAP Kinases Can Trigger Various Phosphorylation Cascades in *S. japonicum*

MAP kinases (MAPKs) can regulate an array of main cellular processes. The MAPKs, as protein-serine/threonine kinases, are evolutionarily conserved cellular regulatory molecules that convert extracellular stimuli in the form of phosphorylation cascades to intracellular responses (Cargnello and Roux, 2011). MAPKs comprise the extracellular signal-regulated kinases (ERKs), the c-Jun amino-terminal kinases (JNKs), and p38. ERKs primarily participate in the control of cell division, JNKs are critical regulators of transcription, and the p38 MAPKs are activated by inflammatory cytokines and environmental stresses and may lead to inflammatory diseases (Johnson and Lapadat, 2002). In recent years, some articles on MAPKs have studied their relationship with schistosomiasis. The research related to *S. mansoni* shows that MAPKs play important roles in normal development, successful survival, and reproduction of the schistosomes, furthermore, ERK and JNK are probably considered as potential targets for drug invention (Andrade et al., 2014). Through analyzing the expression levels of MAPKs mRNAs in different developmental stages of *S. japonicum*, it was found that ERK, JNK, and their inhibitor Sja-DSP are all markedly upregulated in adult female schistosomes, compared with male worms. In particular, ERK was more significantly upregulated in cercariae than other stages of in schistosome. These results suggests that ERK and JNK may participate in female sexual maturation processes, and ERK may also be involved in the growth and development of schistosomes (Wang et al., 2006). In addition, the expression level of muscle Ras oncogene homolog (MRAS) is higher in female worms and eggs than male worms, indicating that they also can regulate normal growth and development of the schistosomes.

**Figure 4:**
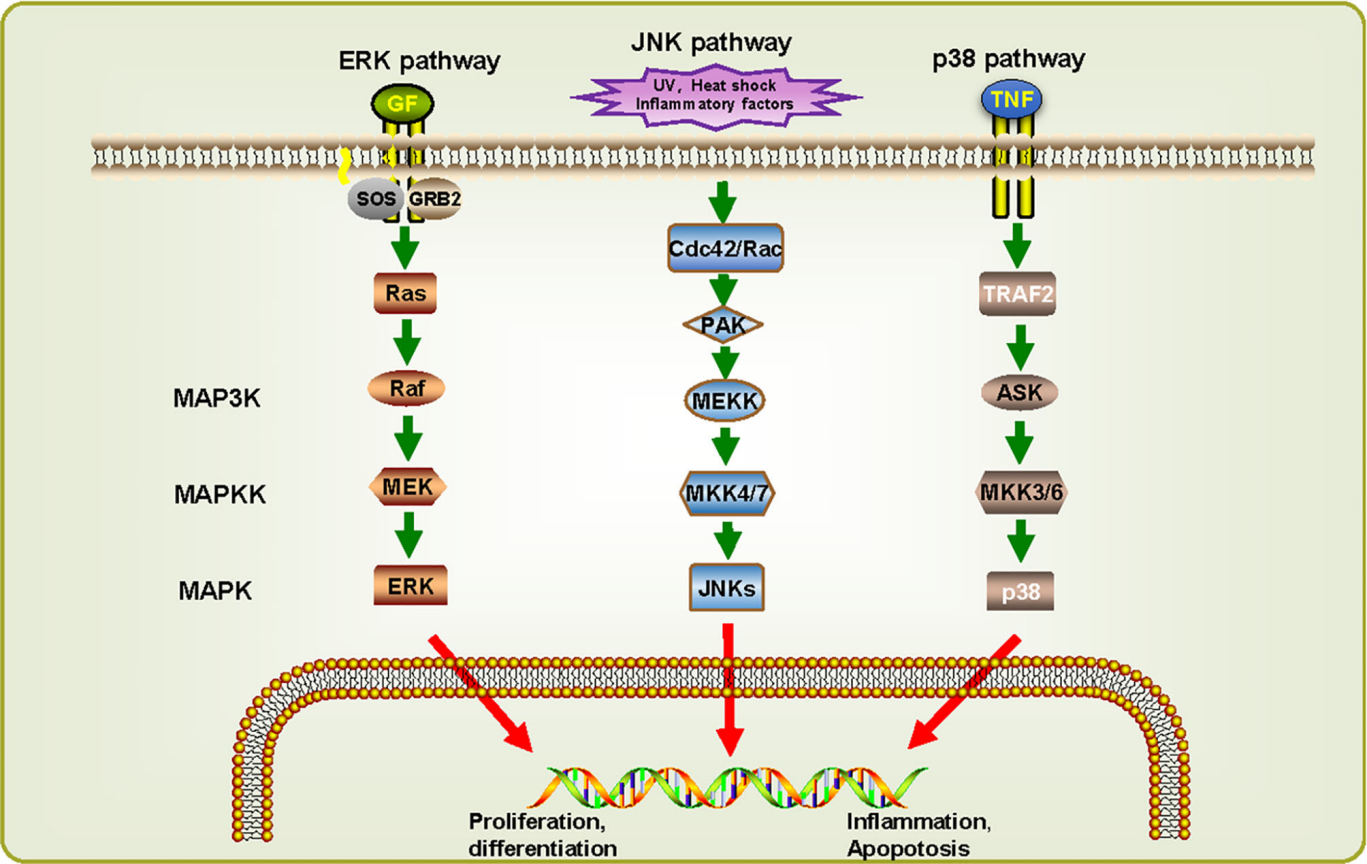
MAPK signaling cascades in *Schistosoma japonicum*. Three signaling pathways were predicted: ERK pathway, JNK pathway, and p38 pathway. They can be classified into three kinase modules including a MAPK, a MAPK kinase (MAP2K/MKK) and a MAPK kinase kinase (MAP3K/MKKK). The proteins shown in white have not (yet) been identified in *S. japonicum*. UVw, heat shock, inflammatory factors, and cellular stresses can advance the activation of different MAPK pathways, which in turn phosphorylate downstream molecules.

By reconstructing the predicted MAPK signaling pathways in *S. japonicum* based on known MAPK signaling pathways (**Figure 4**) (Wang et al., 2006), most components of these pathways are also present in *S. japonicum*, indicating that the MAPK signaling pathways are highly conserved. For ERK pathway, when RTK binds to growth factor (GF), growth factor receptor-bound protein 2 (GRB2) binding site is exposed, and son of sevenless (SOS) is recruited to the cell membrane to activate Ras before it binds to Raf. Then activated Raf phosphorylates MEK, and ERKs are activated. For JNK pathway, UV, heat shock, and inflammatory factors can stimulate the cascades phosphorylation of Cdc42/Rac, PAK, MEKK, MKK4/7, and JNKs. For p38 pathway, it can be activated by the combination of tumor necrosis factor receptor and tumor necrosis factor (TNF). Although there is no p38 sequence detected in *S. japonicum*, the presence of MKK3/6 can be responsible for the activation of p38, suggesting that the p38 pathway might also exist in *S. japonicum*. These observations can demonstrate that MAPK signaling pathways may play a critical role in proliferation, differentiation, growth, inflammation, and apoptosis in *S. japonicum*.

## Atypical Protein Kinases Are Associated With the Growth and Development of *S. japonicum*

It has been confirmed that aPKs exhibit low sequence similarity compared with ePKs, which generally share the archetypical kinase fold but lack conserved eukaryotic kinase motifs and have altered hydrophobic spines (Kanev et al., 2019). Right open reading frame protein kinase 2 (Riok-2) belongs to Rio kinase, which is one of aPKs family and is involved in RNA biogenesis and cell cycle processes (LaRonde-LeBlanc and Wlodawer, 2005a; LaRonde-LeBlanc and Wlodawer, 2005b). Recently, it has been reported that Riok-2 may be related with physical activity in *S. japonicum*. *In silico* analyses of Sj-Riok-2 shows its typical features including a winged-helix (wHTH) domain, a flexible loop, a hinge region, and a metal-binding loop. Transcriptional profiling suggests that Sj-Riok-2 is expressed in all developmental stages of *S. japonicum*, and the transcription level in female worms and eggs is higher than that in male worms. Furthermore, in adult females, Sj-Riok-2 is mainly localized in the vitellarium and ovary, indicating that it can participate in female reproductive biology in *S. japonicum* (Zhao et al., 2017). It has been shown that genes involved in cell cycle processes, nucleotide biosynthesis, amino acid metabolism, DNA synthesis are more significantly up-regulated in female adult worms (Cai et al., 2016; Zhao et al., 2017). This may explain why Sj-Riok-2 is more abundant in adult females and eggs, compared with adult males. The results of pairing experiments reveal that Sj-Riok-2 may participate in pairing-influenced processes related to female maturation and sexual maintenance (Zhao et al., 2017). A previous study has demonstrated that Riok-2 can be phosphorylated by Polo-like kinase 1 (Plk1) to regulate mitotic progression (Liu et al., 2011). Double knockdown of Sj-plk-1 and Sj-Riok-2 induces a significant reduction of EdU-labelled cells in the gonads, and single knockdown of Sj-Riok-2 can reduce cell proliferation in the gonads and increase the number of mature oocytes in the ovary as well as an accumulation of eggs in the uterus (Zhao et al., 2017). Surprisingly, it has not been found that the interference of Rio2 and Plk1 had a significant effect on the transcription level of apoptosis genes, indicating that Riok-2 and Plk1 could participate in oocytes or vitelline cell maturation that does not rely on activating apoptosis pathway. Accordingly, Sj-Riok-2 might play an important role in the development and maturation of *S. japonicum*. In addition, bioinformatics analysis shows that Sj-Riok-2 contains phosphorylation sites and glycosylation sites, therefore, it is speculated that it may be immunogenic, which can contribute to the development of high-efficiency epitope vaccines for schistosomiasis, and provide a new target for the clinical diagnosis and treatment of schistosomiasis (Zhao et al., 2018).

In general, whether Sj-Riok-2 is involved in the maturation and development of schistosomes *in vivo* and whether it can be used as a drug target for schistosomes need further research such as applying corresponding inhibitors and gene knockout.

## Protein Kinase Inhibitors Are Potential Drugs Against *S. japonicum*

On account of the increasing resistance and side effects of PZQ, researchers are seeking for new drugs. Some researches focus on conventional drug that used for treating other diseases. A dose of 150 mg/kg mefloquine, an antimalarials drugs, can cause a significant reduction in *S. mansoni* eggs in infected mice (Van Nassauw et al., 2008), but it has dose-dependent negative effects in children and adults (Basra et al., 2013). Artemisinin is the most potent antimalarial drugs, its derivatives such as artemether and artesunate, have been shown in several studies to have anti-schistosomal activity among three schistosomes, both in human and animal experiments (Utzinger et al., 2000; De Clercq et al., 2002; Li et al., 2005). However, certain experiments are still needed to prove the specific efficacy of artemisinin. Recent studies have discovered some novel drugs such as antimalarial quinoxaline derivative (MMV007204), N,N’-Diarylurea MMV665852 analogs and 15β-senecioyl-oxy-ent-kaur-16 -en-19-oic acid, but their efficacy are not superior to PZQ (Cowan et al., 2015; Sessa et al., 2020; Debbert et al., 2021). Therefore, it is of great significance to develop novel and affordable anti-schistosomiasis drugs that are structurally and functionally different from PZQ in order to tackle the rapid development of PZQ resistance.

PKs have been considered as critical targets for drug intervention, and an increasing number of kinase inhibitors have been used for cancer therapy, the US Food and Drug Administration (FDA) had approved 28 small-molecule kinase inhibitors for therapy by April 2015 (Rask-Andersen et al., 2014; Wu et al., 2015). In schistosomes, the complex and important functions of PKs play important roles in their growth, development, and maturation. It is promising to design protein kinase inhibitors, whether it be treating schistosomiasis alone or in combination with PZQ.

TKs are essential to many cellular processes, so they have been considered as interesting drug targets for treating helminthic diseases (Dissous et al., 2007; Knobloch et al., 2007; O’Connell et al., 2015). Abelson (ABL) family kinases, which belong to TKs, are grouped into ABL1 and ABL2, which can regulate different physiological processes including cell proliferation, survival, migration, and invasion (Greuber et al., 2013; Khatri et al., 2016). ABL kinases have been shown to be the cause of chronic myelogenous leukemia (CML) in humans, and they are also found in the schistosomes genome (Deininger et al., 2000; Berriman et al., 2009). In addition, there are two ABL kinases, SmABL1 and SmABL2, expressed mainly in the ovary, vitellarium, and testes, weakly in the parenchyma and gastrodermis in *S. mansoni* (Beckmann and Grevelding, 2010). Imatinib, an ABL inhibitor, targets the adenosine triphosphate (ATP)-binding site, exerts a negative effect on gonad development and pairing stability in *S. mansoni*, and eventually causes the death of the parasite (Manley et al., 2002; Beckmann and Grevelding, 2010). However, the subsequent *in vivo* experimental results show Imatinib has no influence on worm burden or egg-production, and the reason may be that the mouse is not a suitable infection model for testing the effect of Imatinib *in vivo*. (Beckmann et al., 2014). Imatinib has also been used to study the effect of killing *S. japonicum*. The worms treated with Imatinib showed a significant reduction of motility and pairing stability, and adult worms were killed after 3-5 days in culture (Li et al., 2019). Serious morphological changes including tegument bubbles, tegument detachment, and bulges were also observed. Furthermore, Imatinib not only reduces the numbers of excreted eggs and causes the formation of abnormal eggs, but it also affects gonad development and digestive organs, confirming that Imatinib may be an attractive drug against *S. japonicum*, but more experiments *in vivo* and *in vitro* are needed to prove this hypothesis (Li et al., 2019).

At present, except the above-mentioned Imatinib, there are several anti-cancer drugs designed based on human protein kinases that have potential effects on the treatment of schistosomiasis. Most of them focus on *S. mansoni*, such as Genistein. The study observed that some indicators were significantly reduced including the total worm burden, tissue egg load, hepatic granulomas diameter and areas in mice liver after the application of Genistein (Sobhy et al., 2018). In addition, the best results were observed with the combination of Genistein and PZQ, revealing that Genistein can be a promising complementary therapy to PZQ (Sobhy et al., 2018). Sorafenib, a multiple tyrosine kinase inhibitor, has been confirmed to suppress *S. japonicum*-induced liver fibrosis significantly in mice with the combination of PZQ (Ma et al., 2018). Regrettably, the specific mechanism of action on *S. japonicum* is not elucidated, but it provides a possible way to discover potential PKs inhibitors for schistosome from conventional anti-cancer drugs. Interestingly, a novel computational drug repurposing pipeline has screened a series of tyrosine kinase inhibitors (anti-cancer drugs) which could be new schistosomicidal agents, such as imatinib, bosutinib, crizotinib, nilotinib, and dasatinib (Giuliani et al., 2018). In the future, it will be meaningful to design PKs inhibitors for the treatment of schistosomiasis based on the targets of these anti-cancer drugs, although there is still a long way to go.

## Concluding Remarks and Future Prospects

Due to the complicated transmission routes and many influencing factors of schistosomiasis, it is hard to prevent and control schistosomiasis (Lü et al., 2021). Although schistosomiasis japonica has been basically contained in China, it is still a public health problem in endemic areas in the Philippines and remote areas in Indonesia (Olveda and Gray, 2019). Designing specific targeted drugs and developing safe and effective vaccines are direct methods. Recent progress has confirmed that some PKs are related to the growth, development, and survival of *S. japonicum* (e.g., TK family, GSK, CAMKII, MAP kinases, aPKs). Their locations and functions are summarized and shown in **Table 1**. But the specific regulation mechanism in the worm has not yet been clarified, one reason being that most results are obtained mainly based on *in vitro* experiments, which lack effective and sufficient *in vivo* experimental data to verify. Another important reason is the absence of advanced and effective technology, which hinders further studies. Currently, most investigations are performed based on RNAi and histological observation. However, the sensibility of RNAi varies from gene to gene, and its efficiency depends on the expression level, and tissue location of the target mRNA and protein. A long-term RNAi technology has been described as an economical and effective protocol for the knockdown of target genes in *S. japonicum* (Li et al., 2018), which is a potential tool for future research. Excitingly, the advent of CRISPR (Clustered Regularly Interspaced Short Palindromic Repeats) technology could elevate our understanding of genomic functions, parasite biology, and parasite-host interactions (Du et al., 2021). Furthermore, the deficiency of comprehensive information on *S. japonicum* protein kinase has seriously obstructed the progress in related research, and thus most studies based solely on the discovery of other species of schistosomes.

Recently, high-quality assembly of *S. japonicum* genome has been reported, providing a novel reference genome of *S. japonicum* and beneficial to functional genomic and comparative genomics of schistosome (Luo et al., 2019). In the future, we can construct a corresponding kinase map to explore the interesting targets according to this genome information. The phosphorylation sites may affect the binding effect of the drugs and the targets (Hirst et al., 2020), and widespread phosphorylation data can support the development of kinase drugs. The application of CRISPR technology can allows us to modify the genes of interest, and then functionally analyze the key targets. The advent of artificial intelligence is beneficial to early drug discovery including target identification and validation, virtual screening, drug repurposing, it will be exciting to apply this technique to schistosome drugs screening. With the combination of CRISPR technology and artificial intelligence technology, the development of anti-schistosomiasis drugs will be greatly accelerated. In addition, we can concentrate on studying the host-parasite interaction of PKs, such as the RTK family, which will benefit the design of drug targets and lead a better understanding of the pathogenesis of schistosomiasis. In view of the deficiency of PZQ, combining PZQ with new drugs resist schistosomula is also the future trend of drug selection.

Overall, SjPKs, potential drug targets for treating schistosomiasis japonica, are worthy of further research. Considering that the PKs of *S. japonicum* are homologous and conserved with those of humans, it is better to choose structural regions other than the conserved catalytic domain of PKs, to design kinase targets that exist in schistosomes but not in humans. Progress in the function of PKs also lays the foundation for finding drug targets of other parasites.

## Author Contributions

KW and JH conceived the manuscript. KW collected and reviewed the literature, and wrote the manuscript. KW, XZ, and ZY generated the figures and table. SH and LJ reviewed the literature. JH reviewed and edited its final manuscript. All authors contributed to the article and approved the submitted version.

## Funding

The work was supported by the Natural Science Foundation of Hunan Province (2020JJ5700 to JH, 2020JJ5702 to ZY), and the start-up funding for young talents of Central South University (202045004 to JH).

## Conflict of Interest

The authors declare that the research was conducted in the absence of any commercial or financial relationships that could be construed as a potential conflict of interest.
